# Comparing traditional and online problem-based learning in child and adolescent psychiatry in Nagoya: a novel statistical approach in Japanese educational settings

**DOI:** 10.1186/s12909-026-09517-9

**Published:** 2026-06-10

**Authors:** Basile Chretien, Kazuki Nishida, Takeshi Kondo, Noriyuki Takahashi, Hideki Takami, Hiroshi Nishigori, Branko Aleksic, Charles Dolladille, Itzel Bustos Villalobos, Tetsuya Yagi, Norbert Skokauskas, Hideki Kasuya

**Affiliations:** 1 Department of International Medical Education, Nagoya University Graduate School of Medicine, Nagoya, Japan; 2 Neuropresage Team, Physiopathology and Imaging of Neurological Disorders (PhIND), Institute Blood and Brain @ Caen-Normandie (BB@C), Normandie University, UNICAEN, INSERM, UMR-S U1237, GIP Cyceron, Caen, 14000 France; 3 Unit of Biostatistics, Department of Advanced Medicine, Graduate School of Medicine, Nagoya University, Nagoya, Japan; 4 Center for Postgraduate Clinical Training and Career Development, Nagoya University Hospital, Nagoya, Japan; 5 School of Health Professions Education, Maastricht University, Maastricht, The Netherlands; 6 Department of Education for Community-Oriented Medicine, Nagoya University Graduate School of Medicine, Nagoya, Japan; 7 Center for Medical Education, Nagoya University Graduate School of Medicine, Nagoya, Japan; 8 Department of Psychiatry, Nagoya University Graduate School of Medicine, Nagoya, Japan; 9 Department of Pharmacology, Caen-Normandy University Hospital, Caen, France; 10 Department of Infectious Diseases, Nagoya University Graduate School of Medicine, Nagoya, Japan; 11 Regional Centre for Child and Youth Mental Health and Child Welfare, Department of Mental Health, Norwegian University of Science and Technology, Trondheim, Norway

**Keywords:** Problem-Based Learning, Child and Adolescent Psychiatry, Online Learning, Medical Education, Japan, Cluster Analysis

## Abstract

**Background:**

Problem-Based Learning (PBL) requires active communication and learner autonomy, which can be challenging in cultural contexts that emphasize group harmony and indirect communication, such as Japan. This challenge is often amplified when PBL is conducted in a non-native language (English), potentially inducing “foreign language anxiety.” While the COVID-19 pandemic necessitated a shift to online learning, the specific impact of this format on psychological barriers and student engagement in high-context cultures remains underexplored. We investigated whether the online format could reduce these transactional distances and enhance learning outcomes compared to traditional in-person PBL.

**Methods:**

We conducted a naturalistic, historical control study comparing fourth-year medical students at Nagoya University during a Child and Adolescent Psychiatry curriculum. The 2019 cohort (*n* = 109) participated in-person, while the 2021 cohort (*n* = 100) participated online. We administered a 15-item questionnaire assessing satisfaction, engagement, and case suitability. Beyond standard descriptive comparisons, we applied a novel multidimensional statistical framework. This included Ordered Logistic Regression adjusted for age and sex to identify predictors of satisfaction, Exploratory Factor Analysis (EFA) to validate the instrument’s latent structure, and K-means Clustering to identify distinct student “phenotypes” based on response patterns.

**Results:**

The online cohort reported significantly higher satisfaction across most domains (Odds Ratios 0.36–0.51, *p* < 0.05). Factor analysis identified two primary dimensions—“Perceived Learning Efficacy” and “Engagement”—while “Communication Skills” (Question 4) emerged as an independent outlier, failing to load on either dimension. Cluster analysis identified two distinct student phenotypes: a “Traditional/Dissatisfied” profile (Cluster 1: *n* = 77, 69% from the in-person group, predominantly male and older) and a “Digital/Satisfied” profile (Cluster 2: *n* = 127, 57% from the online group, predominantly female and younger).

**Conclusions:**

Online delivery of PBL was associated with significantly higher student satisfaction and engagement scores in this Japanese context. Because the study is observational (historical-cohort comparison with no randomisation), this association cannot be attributed causally to the modality, and the role of reduced foreign-language anxiety is an interpretive hypothesis that the present design does not directly test. The identification of distinct student phenotypes suggests that demographic factors (gender, age) and delivery modality interact to shape the learning experience. These findings advocate for a tailored pedagogical approach, where digital formats serve as a “safe harbor” for students with high communication apprehension.

**Supplementary Information:**

The online version contains supplementary material available at 10.1186/s12909-026-09517-9.

## Background

Japan is currently navigating profound demographic challenges that are reshaping the social and healthcare landscape. With one of the world’s most rapidly aging populations and a consistently declining birth rate, the country is experiencing a demographic inversion that affects every facet of society [1]. The younger generation is shrinking, creating fewer caregivers for the elderly, while the “sandwich generation” of adults is burdened with dual responsibilities: supporting their aging parents and raising their children [2]. These circumstances create a ripple effect on family dynamics and place significant pressure on children and adolescents, who are often caught in the crossfire of these societal and familial stressors [3].

At the same time, modern stressors such as academic pressures, cyberbullying, and the pervasive influence of social media are shaping the mental health landscape of young people [4–6]. Although the absolute number of children in Japan is declining [7], mental health concerns among this population are becoming more prominent [8]. Addressing these challenges requires a robust healthcare system staffed with professionals who are not only clinically skilled but also attuned to the unique sociocultural factors influencing mental health in Japan. A significant barrier to mental healthcare in Japan has historically been the stigma surrounding mental illness [9]. However, societal attitudes are gradually shifting, driven by public health campaigns and a growing recognition of mental health as a critical component of overall well-being [10].

For medical students, early and effective education in child and adolescent psychiatry (CAP) is essential. In Japan, Problem-Based Learning (PBL) has been heralded as a transformative model to bridge the gap between theoretical knowledge and practical application [11]. Unlike traditional lecture-based methods, PBL engages students in active learning through the exploration of real-world clinical scenarios, fostering critical thinking, problem-solving, and collaboration [12]. Since its introduction to Japan in 1990, PBL has been widely adopted [13, 14], including at Nagoya University (NU) [15], where it is integrated into the fourth-year curriculum with specific applications in CAP [16]. NU has also partnered with international institutions such as the Norwegian University of Science and Technology (NTNU) to refine its PBL cases and incorporate cross-cultural perspectives, particularly through initiatives like the TroNa Partnership and Equal Partnership.

However, the effectiveness of PBL relies heavily on active communication and student autonomy, requirements that may conflict with specific cultural and linguistic traits of Japanese students. Previous studies have shown that Japanese students tend to have limited proficiency in English [17, 18] and a cultural tendency toward shyness, particularly when speaking foreign languages [19]. When PBL is conducted in English, these factors may generate significant anxiety and cognitive load, potentially hindering engagement in a traditional face-to-face setting.

To understand how these barriers might be mitigated, we turn to Transactional Distance Theory (TDT) [20] and the Community of Inquiry (CoI) [21] framework. TDT posits that “distance” in education is not merely geographic but psychological and pedagogical, determined by the interplay of dialogue, structure, and learner autonomy. For Japanese medical students, the pressure of immediate, face-to-face English interaction may paradoxically increase this “transactional distance,” leading to withdrawal. Conversely, online environments—often criticized for lacking social presence—may offer a “safe harbor” for these students. By providing a digital buffer and allowing more time for cognitive processing, the online format could reduce the psychological distance and communication apprehension predicted by TDT. Similarly, the CoI framework suggests that effective learning requires the intersection of social, cognitive, and teaching presence. We hypothesize that for this specific demographic, the online modality may enhance cognitive presence by lowering the social anxiety associated with in-person English performance.

Despite the growing body of literature on PBL, many studies focus on simple descriptive outcomes and fail to account for these complex cultural and theoretical interactions. Understanding these underlying patterns is essential to optimize educational strategies for diverse student populations. This study builds on previously published data on PBL in CAP at NU [22], using advanced statistical methods like adjusted logistic regression (specifically, ordered logistic regression adjusted for age and sex; see Methods) and factorial analyses to uncover patterns that are not immediately apparent [23, 24].

The goal of this post-hoc analysis is to compare the traditional, in-person PBL method conducted in English with an online PBL method, also in English, among Japanese medical students. Guided by TDT and CoI, we aim to determine whether the online format—by potentially reducing the transactional distance created by language and cultural barriers—is more suitable for this population. Through this study, we aim to enhance the understanding of PBL’s role in medical education, contributing to the preparation of a healthcare workforce equipped to meet the challenges of the future.

Specifically, the primary aim of this study is to compare overall student satisfaction with PBL between the 2019 in-person cohort and the 2021 online cohort, after adjustment for age and sex. The secondary aims are exploratory and psychometric: (i) to describe pairwise correlations between items overall and within each cohort; (ii) to characterise the dimensional structure and reliability of the questionnaire by exploratory factor analysis with formal factorability indices (KMO [25] and Bartlett’s [26] test) and by confirmatory factor analysis using an ordinal estimator; (iii) to compare cohort-level composite scores on the retained dimensions; and (iv) to identify, via k-means clustering, response-pattern subgroups (“student phenotypes”) and the demographic and modality features associated with them. The modality comparison is the only confirmatory question of the study; the psychometric and clustering analyses are exploratory and hypothesis-generating.

## Methods

### Study design and setting

We conducted a cross-sectional, naturalistic study comparing two distinct cohorts of fourth-year medical students at Nagoya University School of Medicine. The study utilizes a historical control design to evaluate the impact of the delivery modality on student satisfaction and engagement in a Child and Adolescent Psychiatry (CAP) Problem-Based Learning (PBL) module. The control group consisted of students who completed the traditional in-person PBL curriculum in 2019. The intervention group consisted of students who completed the same curriculum in an online synchronous format in 2021, following the university’s transition to remote learning due to the COVID-19 pandemic.

#### Participants and sampling

The target population included all fourth-year medical students rotating through the CAP department during the respective academic years. No exclusion criteria were applied based on age, gender, or nationality. As this was a curriculum-wide educational evaluation, total population sampling was used rather than random sampling. A total of 112 students participated in 2019, and 111 students participated in 2021. This sampling strategy is therefore a convenience, non-probability sample of a complete academic cohort. No a-priori sample-size calculation was performed: as the present work is a post-hoc analysis of a fixed pre-existing dataset, the sample reflects the entire eligible population for each year. Missing values were rare (between one and four missing entries per item out of 209 respondents) and were handled by complete-case analysis within each model; descriptive tables report the number of non-missing observations for each variable.

### The PBL intervention

Both cohorts followed an identical pedagogical structure involving a “Mayumi” case scenario (S Data 1), which details the clinical presentation of a 15-year-old girl with depression. The module consisted of two 90-minute sessions facilitated by faculty tutors. Students were divided into small groups of approximately 10 participants, with assigned roles including a group leader and a note-taker.

- In-Person Format (2019): Sessions were conducted in physical seminar rooms. Discussions relied on whiteboards for brainstorming and face-to-face interaction.
- Online Format (2021): Sessions were conducted synchronously via video conferencing software (Microsoft Teams^®^). Breakout rooms were utilized for small-group discussions, and digital shared documents were used in place of physical whiteboards.

In both formats, the case materials were provided in English to align with the university’s internationalization goals. However, to reduce cognitive load and facilitate complex clinical reasoning, students were permitted to conduct discussions in either English or Japanese (code-switching).

### Data collection and instrument

Immediately following the PBL sessions, students were invited to complete a voluntary, anonymous 15-item questionnaire (S Table 1). The instrument was developed by faculty experts in medical education and psychiatry to capture three specific theoretical domains: The instrument was adapted from the questionnaire used by Iwatsuki et al. (2021) on the same Nagoya CAP PBL cohort [22], which had in turn been informed by Kalaian and Mullan’s exploratory factor analysis of PBL ratings [24]. Items were reviewed for face and content validity by four Nagoya University Graduate School of Medicine faculty members with combined expertise in child and adolescent psychiatry, medical education and biostatistics. No separate pilot study with formal reliability testing was conducted before the 2019 administration; the implications of this are discussed in the Strengths and Limitations section, and the psychometric properties reported below should be regarded as part of the on-going validation of the instrument rather than as confirmation of pre-established validity.

1. General PBL Perception (Q1–Q5): Assessing the acquisition of self-directed learning and clinical problem-solving skills.
2. Subject Interest (Q6–Q10): Assessing engagement specifically with the field of Child and Adolescent Psychiatry.
3. Case Suitability (Q11–Q15): Evaluating the realism, difficulty, and educational value of the specific “Mayumi” scenario.

Items were scored on a three-point Likert scale (1 = Agree, 2 = Neutral, 3 = Disagree). While a wider scale is common in psychometrics, a three-point scale was selected to minimize respondent burden and central tendency bias in this specific student population. The instrument was treated as exploratory; therefore, internal consistency and structural validity were assessed post-hoc as part of this study’s analysis. Because items were coded with 1 = Agree and 3 = Disagree, lower scores correspond to higher satisfaction; as a corollary, item-level odds ratios below 1 in the online group and a higher composite score in the in-person group are mutually consistent and both indicate greater satisfaction in the online cohort.

### Statistical analysis

All statistical analyses were performed using R software (version 4.4.1), utilizing the psych and lavaan packages. Statistical significance was set at *p* ≤ 0.05.

#### Descriptive and bivariate analysis

Categorical variables were summarized as counts and percentages, and continuous variables (age) as medians with interquartile ranges (IQR). Differences in demographic characteristics between the in-person and online groups were assessed using Pearson’s Chi-squared test or Fisher’s exact test for categorical variables, and the Wilcoxon rank-sum test for age.

#### Comparative analysis (outcomes)

To evaluate the primary outcome (student satisfaction), we employed Ordered (proportional-odds) Logistic Regression. This method is appropriate for ordinal data (Likert scales) and allows for multivariate adjustment. Models were constructed for each individual survey item, assessing the impact of the PBL modality (Online vs. In-person) while adjusting for potential confounders including age and sex. Additionally, a composite satisfaction score was calculated (sum of all items; range 15–45) and compared between groups using multiple linear regression, adjusted for age and sex. The composite-score comparison was pre-specified as the single confirmatory test of the modality effect; the 15 item-level ordered-logistic models were exploratory and intended to localise which questionnaire items drove the global pattern. Bonferroni and Benjamini-Hochberg corrections were considered but were not imposed on this exploratory layer, because (i) doing so would conflict with its hypothesis-generating role, and (ii) the per-family Bonferroni threshold here (alpha = 0.05/15 = 0.003) is exceeded by the majority of reported item-level p-values, so the substantive interpretation is unchanged; readers wishing to apply a strict family-wise correction can re-evaluate Table 3 against that threshold.

#### Structural validity (factor analysis)

To address the construct validity of the instrument, we first assessed the factorability of the 15-item correlation matrix with the Kaiser-Meyer-Olkin (KMO) measure of sampling adequacy [25] and Bartlett’s test of sphericity [26]. The number of factors to retain was decided by combining scree-plot inspection, the Kaiser criterion (eigenvalue > 1) and Horn’s parallel analysis (psych::fa.parallel) [27] rather than by any single rule. Exploratory Factor Analysis (EFA) was performed using principal axis factoring with varimax (orthogonal) rotation; varimax was chosen a-priori to obtain an interpretable simple structure under the working hypothesis that the two main dimensions of the questionnaire, perceived learning efficacy and case-specific appropriateness, would be largely independent. Internal consistency was evaluated with Cronbach’s alpha [28] for the full scale and for each retained factor. The two-factor model was then re-estimated by confirmatory factor analysis (CFA) using the WLSMV estimator with ordered indicators in lavaan, which is the recommended estimator for ordinal Likert items with few response categories [29].

#### Student profiling (cluster analysis)

To identify distinct phenotypes of student engagement, we performed k-means clustering. The optimal number of clusters was determined using the Within-Cluster Sum of Squares (WSS) elbow method and silhouette analysis. This unsupervised learning approach allowed us to move beyond average scores and identify subgroups of students with similar response patterns (e.g., “High Satisfaction” vs. “Dissatisfied”) regardless of their cohort.

### Ethical considerations

This study was conducted in accordance with the ethical standards of Nagoya University and the 1964 Declaration of Helsinki and its later amendments. The study follows a retrospective design utilizing data originally collected as part of a routine educational evaluation of the PBL program. Participation in the questionnaire was voluntary, and students were informed that their anonymous responses might be used for quality improvement and future academic purposes. The consent process varied by cohort to accommodate the delivery format: for the 2019 in-person group, verbal consent was obtained, and agreement was documented through the voluntary submission of the completed questionnaire; for the 2021 online group, written informed consent was obtained electronically. The Nagoya University Ethical Committee retrospectively reviewed and approved the study protocol, including the use of this pre-existing data and the respective consent procedures, on March 5th, 2022 (Approval Code: 2021 − 0482). The dataset was subsequently accessed for research analysis on March 22nd, 2022.

## Results

**Fig. 1:**
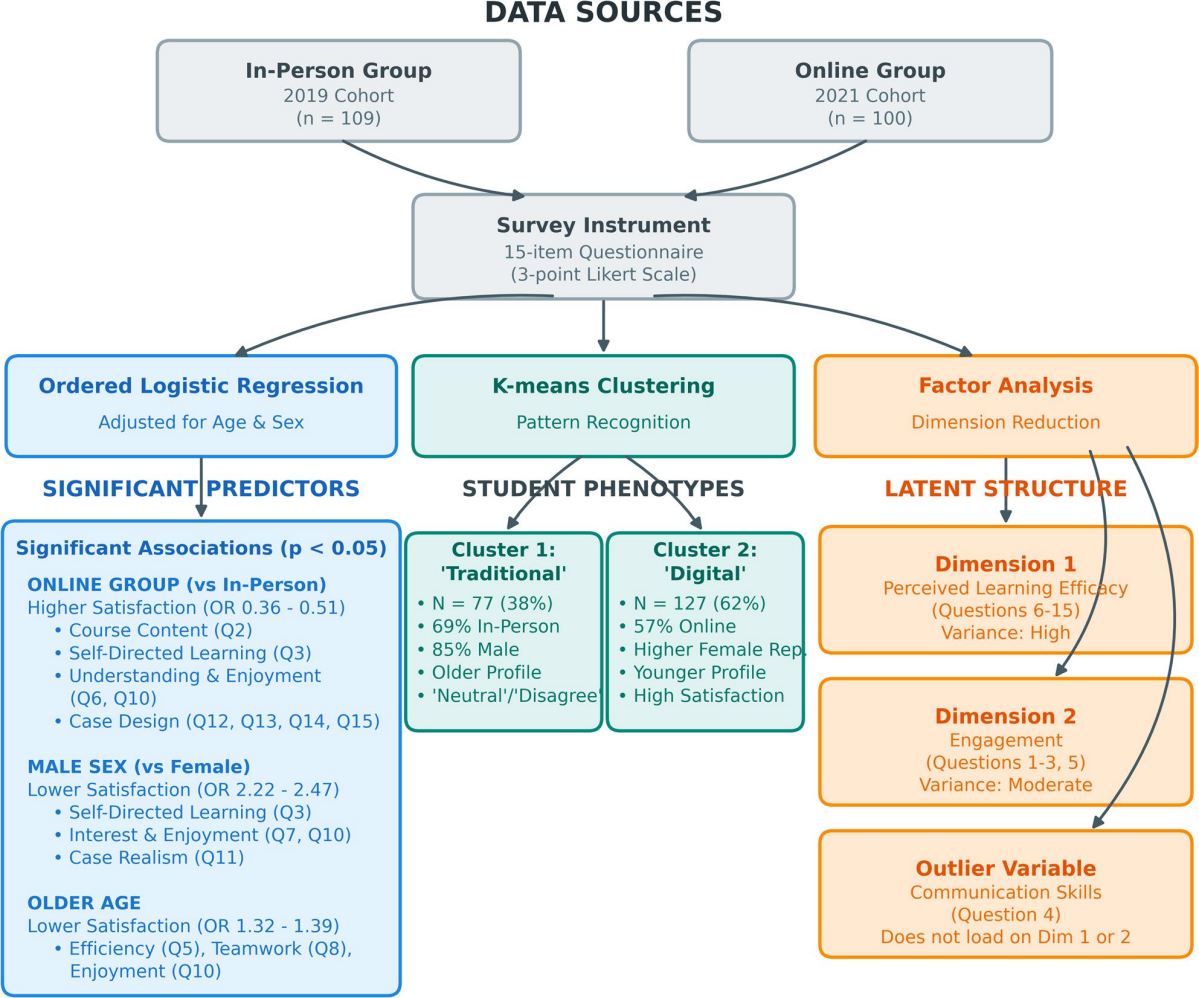
Study flowchart illustrating the analytical framework, statistical methods, and summary of key findings (student phenotypes)

Of the 112 students who participated in the in-person PBL, 109 completed the survey, while among the 111 participants in online setting, 100 participants filled out the questionnaire. Age ranged from 21 to 32 years old and was not statistically different among the 2 groups with a median of 22 years old for both groups. The overall analytical framework, including participant flow, statistical methods, and a summary of key findings (significant predictors and student phenotypes), is presented in Fig. 1.

Males were more represented than females in the 2 groups (76% in the in-person group and 73% in the online group) which was not statistically different. With the exception of one Chinese student in the online group, the cohort was entirely Japanese. (Table 1). Reliability analysis yielded a Cronbach’s alpha of 0.92, indicating excellent internal consistency across the 15-item questionnaire.

**Table 1:** Demographics

PBL type	*N*	in-person *N* = 109^*1*^	online *N* = 100^*1*^	*p*-value^2^
Age	191	22.00 (22.00, 23.00)	22.00 (22.00, 23.00)	0.6
Unknown		11	7	
Gender	189			0.6
F		23 (24%)	25 (27%)	
M		74 (76%)	67 (73%)	
Unknown		12	8	
Nationality	202			0.5
1		109 (100%)	92 (99%)	
2 (Chinese)		0 (0%)	1 (1.1%)	
Unknown		0	7	

^*1*^Median (Q1, Q3); n (%)

^*2*^Wilcoxon rank sum test; Pearson’s Chi-squared test; Fisher’s exact test

Questionnaire results are shown in Table 2, and students in the online group gave a significantly better appreciation of the PBL compared to the in-person group (Table 2). Similar results are found with the ordered logistic regression after adjusting on age and sex (Table 3).

**Table 2:** Answers to the satisfaction questionnaire after PBL

PBL type	*N*	in-person *N* = 109^*1*^	online *N* = 100^*1*^	*p*-value^2^
Q1 : PBL helps me to develop skills to solve clinical problems	208			0.078
1		89 (82%)	91 (92%)	
2		17 (16%)	6 (6.1%)	
3		3 (2.8%)	2 (2.0%)	
Unknown		0	1	
Q2 : PBL is a good way of learning the content of the course	208			0.034
1		77 (71%)	85 (86%)	
2		27 (25%)	12 (12%)	
3		5 (4.6%)	2 (2.0%)	
Unknown		0	1	
Q3 : PBL encourages my self-directed learning	208			0.040
1		71 (65%)	80 (81%)	
2		33 (30%)	17 (17%)	
3		5 (4.6%)	2 (2.0%)	
Unknown		0	1	
Q4 : PBL helps to develop my communication skills	208			> 0.900
1		75 (69%)	66 (67%)	
2		28 (26%)	28 (28%)	
3		6 (5.5%)	5 (5.1%)	
Unknown		0	1	
Q5 : I think, PBL is more effective way of learning compared to traditional lecture	208			0.600
1		71 (65%)	71 (72%)	
2		33 (30%)	24 (24%)	
3		5 (4.6%)	4 (4.0%)	
Unknown		0	1	
Q6 : PBL enhanced my understanding about child & adolescent psychiatry	208			0.001
1		58 (53%)	76 (77%)	
2		40 (37%)	20 (20%)	
3		11 (10%)	3 (3.0%)	
Unknown		0	1	
Q7 : PBL increased my interest in child & adolescent psychiatry	208			0.300
1		60 (55%)	65 (66%)	
2		36 (33%)	26 (26%)	
3		13 (12%)	8 (8.1%)	
Unknown		0	1	
Q8 : Child and adolescent psychiatry because of it’s emphasis on teamwork and collaboration, would be a specialty learned optimally through PBL	208			0.092
1		48 (44%)	56 (57%)	
2		50 (46%)	39 (39%)	
3		11 (10%)	4 (4.0%)	
Unknown		0	1	
Q9 : Duration of PBL session was enough to learn about child & adolescent psychiatry	208			0.300
1		50 (46%)	56 (57%)	
2		41 (38%)	32 (32%)	
3		18 (17%)	11 (11%)	
Unknown		0	1	
Q10 : I enjoyed learning about child and adolescent psychiatry via PBL	205			< 0.001
1		48 (44%)	68 (71%)	
2		47 (43%)	22 (23%)	
3		14 (13%)	6 (6.3%)	
Unknown		0	4	
Q11 : I think Mayumi’s case is common for a clinical-settings in Japan.	208			0.500
1		61 (56%)	57 (58%)	
2		40 (37%)	31 (31%)	
3		8 (7.3%)	11 (11%)	
Unknown		0	1	
Q12 : Mayumi’s case was well written and understandable	208			0.004
1		60 (55%)	76 (77%)	
2		40 (37%)	20 (20%)	
3		9 (8.3%)	3 (3.0%)	
Unknown		0	1	
Q13 : Mayumi’s case has an interesting clinical trigger	208			0.002
1		44 (40%)	64 (65%)	
2		53 (49%)	29 (29%)	
3		12 (11%)	6 (6.1%)	
Unknown		0	1	
Q14 : Mayumi’s case has an appropriate level of difficulty/challenge	208			0.002
1		58 (53%)	76 (77%)	
2		43 (39%)	20 (20%)	
3		8 (7.3%)	3 (3.0%)	
Unknown		0	1	
Q15 : Mayumi’s case helped me to understand the importance of multidisciplinary approach in child and adolescent psychiatry	207			0.009
1		62 (57%)	75 (76%)	
2		39 (36%)	23 (23%)	
3		7 (6.5%)	1 (1.0%)	
Unknown		1	1	

^*1*^Median (Q1, Q3); n (%)

^*2*^Wilcoxon rank sum test; Pearson’s Chi-squared test; Fisher’s exact test

**Table 3:** Ordered logistic regression adjusted on age and sex in order to compare the satisfaction responses between groups to the questionnaire about PBL

Predictors	factor(Q1)			factor(Q2)			factor(Q3)			factor(Q4)			factor(Q5)		
	Odds Ratios	CI	*p*	Odds Ratios	CI	*p*	Odds Ratios	CI	*p*	Odds Ratios	CI	*p*	Odds Ratios	CI	*p*
in-person	*Reference*			*Reference*			*Reference*			*Reference*			*Reference*		
online	0.46	0.18–1.10	0.094	0.43	0.20–0.89	**0.027**	0.51	0.26–0.98	**0.047**	1.18	0.65–2.17	0.587	0.78	0.41–1.46	0.441
Age	1.04	0.76–1.33	0.778	1.21	0.99–1.47	0.055	1.02	0.81–1.25	0.831	0.93	0.73–1.13	0.483	1.39	1.15–1.70	**0.001**
F	*Reference*			*Reference*			*Reference*			*Reference*			*Reference*		
M	1.38	0.52–4.36	0.550	1.70	0.72–4.51	0.250	2.34	1.05–5.79	**0.049**	1.11	0.57–2.25	0.761	1.30	0.64–2.79	0.478
Observations	188			188			188			188			188		

	factor(Q6)			factor(Q7)			factor(Q8)			factor(Q9)			**factor(Q10)**		
*Predictors*	*Odds Ratios*	*CI*	*p*	*Odds Ratios*	*CI*	*p*	*Odds Ratios*	*CI*	*p*	*Odds Ratios*	*CI*	*p*	*Odds Ratios*	*CI*	*p*
in-person	*Reference*			*Reference*			*Reference*			*Reference*			*Reference*		
online	0.36	0.19–0.67	**0.002**	0.68	0.38–1.22	0.203	0.65	0.37–1.15	0.141	0.71	0.41–1.22	0.218	0.36	0.20–0.66	**0.001**
Age	1.10	0.91–1.33	0.306	1.15	0.97–1.37	0.097	1.32	1.10–1.61	**0.004**	1.14	0.95–1.36	0.153	1.34	1.11–1.63	**0.002**
F	*Reference*			*Reference*			*Reference*			*Reference*			*Reference*		
M	1.98	0.96–4.31	0.074	2.22	1.12–4.67	**0.029**	1.72	0.89–3.38	0.113	1.32	0.71–2.51	0.383	2.35	1.17–4.94	**0.021**
Observations	188			188			188			188			185		

	factor(Q11)			factor(Q12)			factor(Q13)			factor(Q14)			**factor(Q15)**		
*Predictors*	*Odds Ratios*	*CI*	*p*	*Odds Ratios*	*CI*	*p*	*Odds Ratios*	*CI*	*p*	*Odds Ratios*	*CI*	*p*	*Odds Ratios*	*CI*	*p*
in-person	*Reference*			*Reference*			*Reference*			*Reference*			*Reference*		
online	1.07	0.60–1.90	0.824	0.37	0.19–0.69	**0.003**	0.45	0.25–0.80	**0.008**	0.37	0.20–0.69	**0.002**	0.51	0.27–0.94	**0.035**
Age	1.17	0.98–1.40	0.083	1.18	0.97–1.43	0.097	1.17	0.97–1.41	0.099	1.08	0.89–1.31	0.416	1.04	0.83–1.29	0.700
F	*Reference*			*Reference*			*Reference*			*Reference*			*Reference*		
M	2.47	1.24–5.20	**0.014**	1.16	0.57–2.45	0.688	0.84	0.44–1.59	0.580	1.70	0.82–3.71	0.170	1.85	0.89–4.11	0.116
Observations	188			188			188			188			187		

**Fig. 2:**
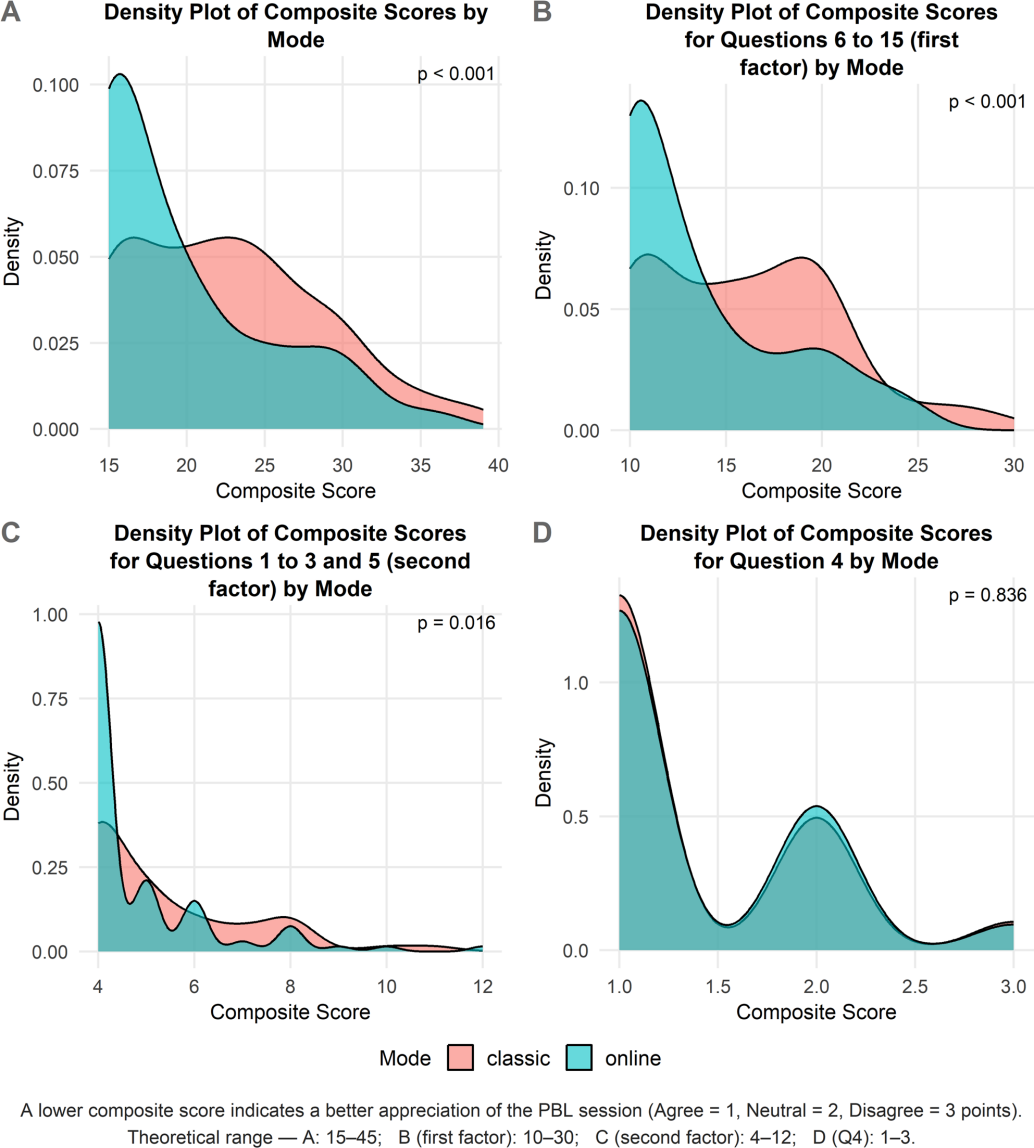
Density plot of Composite score by mode (**A** : for the whole questionnaire, **B** : for the 1st dimension by Mode, **C** : for the 2nd dimension by Mode, **D** : for the 4th question)

Results of the composite score are presented in Fig. 2A: the composite score was statistically higher in the in-person group than in the online group in the multiple linear regression (*p* = 0.02; Table 4). Because the Likert scale is reverse-coded (1 = Agree, 3 = Disagree), a higher composite indicates lower satisfaction, so the composite-score and item-level analyses converge on the same conclusion of greater satisfaction in the online cohort. Since the original data did not follow a normal distribution (skewness = 0.77, kurtosis = 2.71), we acknowledge a potential violation of the normality assumption. However, the residuals from the linear regression were approximately normal (skewness = 0.77, kurtosis = 3.02), supporting the validity of the analysis under the assumption of normal residuals (S Fig. 1). Visual inspection of the residuals versus fitted values revealed a random scatter around zero, with no obvious patterns such as funnel shapes, further supporting the assumption of homoscedasticity. Despite the non-normality of the original data, multiple linear regression remains a robust method in this context [30]. Age was significantly associated with a higher composite score, indicating lower satisfaction among older students. A trend toward higher scores in males was also observed, though not statistically significant. Additionally, a higher proportion of students in the online group reported very high appreciation of PBL, whereas the in-person group had a lower proportion of highly satisfied students.

**Table 4:** Result of the multiple linear regression to compare the composite score between the two groups

Variable	Estimate	95%CI	*p*-value
In-person (reference)	-	-	-
Online	-2.66	[-4.36 ; -0.97]	0.002
Age	0.64	0.08–1.20	0.025
F (reference)	-	-	-
M	1.90	[-0.03 ; 3.83]	0.053

**Fig. 3:**
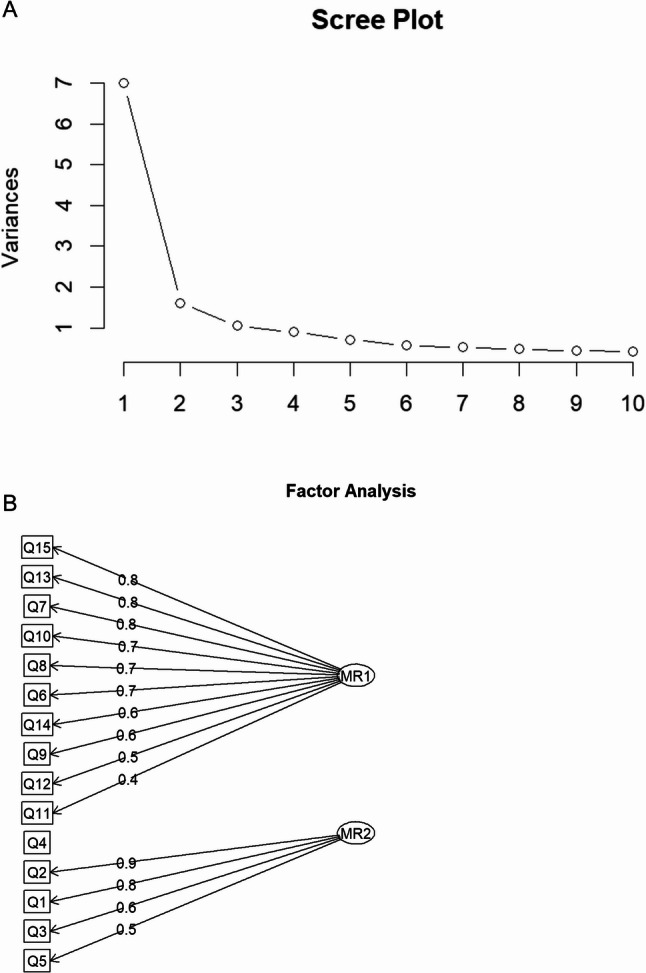
Factor analysis (**A** : Scree plot, **B** : diagram)

When exploring the relationship between different questions, the pairwise scatter plots revealed a consistent response pattern among students. Specifically, students who selected a high rating (e.g., 1) on the Likert scale for one question tended to give similarly high ratings to other related questions. This pattern suggests a strong correlation between certain responses (S Figs. 2 and 3, and 4).

The correlation matrix found high correlations between question 1 to 3, 6 to 10 and 12 to 15. Lower correlations were found between question 4 and the other questions, as well as between question 11 and the other questions. A negative correlation between question 4 and question 12 was found only in the in-person group (S Figs. 5, 6 and 7).

The 15-item correlation matrix was clearly factorable: KMO = 0.90 (Bartlett’s test of sphericity: chi-squared(105) = 1687.9, *p* < 0.001). The first two factors stood well above the random eigenvalues generated by parallel analysis (factor 1: actual eigenvalue 6.47 vs. simulated 95th percentile 0.62; factor 2: 0.97 vs. 0.40), whereas the third factor was only marginally above the threshold (0.38 vs. 0.32) and the fourth was essentially indistinguishable from random (0.25 vs. 0.25). Consistent with this, parallel analysis was sensitive to the number of resampling iterations: with the package default (20 iterations) the procedure returned three factors in 38 of 50 repeated runs, whereas with 500 iterations the procedure stabilised on a two-factor solution. The two-factor solution was therefore retained for the main analyses; the third factor was judged unstable. Internal consistency was good for the overall scale (Cronbach’s alpha = 0.92) and for both retained factors (alpha = 0.91 for the Perceived Learning Efficacy factor [Q6-Q15] and alpha = 0.81 for the case-related/General Impressions factor [Q1, Q2, Q3, Q5]). A scree plot was done in order to assess the number of dimensions for the factor analysis (Fig. 3A). A factor analysis was done for 2 dimensions (Table 5), and questions 6 to 15 were associated to the first dimension, while questions 1, 2, 3 and 5 were associated to the second dimension. Question 4 was not associated with any dimension (Fig. 3B). Confirmatory factor analysis using the WLSMV estimator with ordered indicators supported the two-factor structure identified in the exploratory analysis, with strong standardized loadings for both dimensions and acceptable to good fit indices (CFI = 0.98, TLI = 0.98, RMSEA = 0.074 [90% CI 0.058–0.090], SRMR = 0.07). The composite score based on the factor analysis for the first dimension was also significantly different between the 2 groups. The density plot also found a high satisfaction of the students on the online group compared to the in-person group (Fig. 2B). A similar result was observed for the second dimension (Fig. 2C), while almost no difference could be observed between the groups for the question 4 (Fig. 2D).

**Table 5:** Results of the Factor Analysis

Variable	Factor 1 Loadings	Factor 2 Loadings	Uniqueness
Q1	0.1449725	0.7782357	0.3733321
Q2	0.1722254	0.8676928	0.2174476
Q3	0.3021317	0.6236074	0.5198303
Q4	0.2992293	0.2952419	0.8232940
Q5	0.3675036	0.5017134	0.6132247
Q6	0.7291871	0.3714524	0.3303093
Q7	0.7554548	0.2605440	0.3614049
Q8	0.7321041	0.2964379	0.3761482
Q9	0.6294869	0.3653332	0.4702779
Q10	0.7414557	0.3032310	0.3582943
Q11	0.4438675	0.2187698	0.7551214
Q12	0.5298896	0.1904392	0.6829499
Q13	0.7573570	0.2227423	0.3767963
Q14	0.6467732	0.0850467	0.5744515
Q15	0.7577603	0.1735414	0.3956827

The key characteristics of each cluster are presented in Table 6. The cluster plot indicated a good separation of the students (S Fig. 8). Cluster 1 is composed of 77 students, and is mostly composed of students belonging to the in-person group (69%), and is also composed of 85% of male students and present high proportions of “neutral” or “disagree” answers, indicating a dissatisfaction of the students of this group toward PBL.

**Table 6:** Results of the clustering analysis

By Cluster	*N*	1 *N* = 77^*1*^	2 *N* = 127^*1*^	*p*-value^2^
Mode	204			< 0.001
in-person		53 (69%)	55 (43%)	
online		24 (31%)	72 (57%)	
Age	186	22.00 (22.00, 23.00)	22.00 (21.50, 23.00)	0.006
Unknown		7	11	
Gender	184			0.007
F		10 (15%)	38 (33%)	
M		58 (85%)	78 (67%)	
Unknown		9	11	
Nationality	197			> 0.900
1		76 (100%)	120 (99%)	
2 (Chinese)		0 (0%)	1 (0.8%)	
Unknown		1	6	
Q1 : PBL helps me to develop skills to solve clinical problems	204			< 0.001
1		54 (70%)	123 (97%)	
2		18 (23%)	4 (3.1%)	
3		5 (6.5%)	0 (0%)	
Q2 : PBL is a good way of learning the content of the course	204			< 0.001
1		41 (53%)	118 (93%)	
2		29 (38%)	9 (7.1%)	
3		7 (9.1%)	0 (0%)	
Q3 : PBL encourages my self-directed learning	204			< 0.001
1		35 (45%)	115 (91%)	
2		35 (45%)	12 (9.4%)	
3		7 (9.1%)	0 (0%)	
Q4 : PBL helps to develop my communication skills	204			< 0.001
1		38 (49%)	102 (80%)	
2		30 (39%)	23 (18%)	
3		9 (12%)	2 (1.6%)	
Q5 : I think, PBL is more effective way of learning compared to traditional lecture	204			< 0.001
1		28 (36%)	113 (89%)	
2		42 (55%)	12 (9.4%)	
3		7 (9.1%)	2 (1.6%)	
Q6 : PBL enhanced my understanding about child & adolescent psychiatry	204			< 0.001
1		11 (14%)	119 (94%)	
2		52 (68%)	8 (6.3%)	
3		14 (18%)	0 (0%)	
Q7 : PBL increased my interest in child & adolescent psychiatry	204			< 0.001
1		13 (17%)	111 (87%)	
2		44 (57%)	15 (12%)	
3		20 (26%)	1 (0.8%)	
Q8 : Child and adolescent psychiatry because of it’s emphasis on teamwork and collaboration, would be a specialty learned optimally through PBL	204			< 0.001
1		4 (5.2%)	99 (78%)	
2		60 (78%)	26 (20%)	
3		13 (17%)	2 (1.6%)	
Q9 : Duration of PBL session was enough to learn about child & adolescent psychiatry	204			< 0.001
1		7 (9.1%)	98 (77%)	
2		44 (57%)	26 (20%)	
3		26 (34%)	3 (2.4%)	
Q10 : I enjoyed learning about child and adolescent psychiatry via PBL	204			< 0.001
1		7 (9.1%)	108 (85%)	
2		50 (65%)	19 (15%)	
3		20 (26%)	0 (0%)	
Q11 : I think Mayumi’s case is common for a clinical-settings in Japan.	204			< 0.001
1		19 (25%)	98 (77%)	
2		45 (58%)	26 (20%)	
3		13 (17%)	3 (2.4%)	
Q12 : Mayumi’s case was well written and understandable	204			< 0.001
1		23 (30%)	112 (88%)	
2		44 (57%)	13 (10%)	
3		10 (13%)	2 (1.6%)	
Q13 : Mayumi’s case has an interesting clinical trigger	204			< 0.001
1		8 (10%)	99 (78%)	
2		55 (71%)	27 (21%)	
3		14 (18%)	1 (0.8%)	
Q14 : Mayumi’s case has an appropriate level of difficulty/challenge	204			< 0.001
1		27 (35%)	106 (83%)	
2		40 (52%)	20 (16%)	
3		10 (13%)	1 (0.8%)	
Q15 : Mayumi’s case helped me to understand the importance of multidisciplinary approach in child and adolescent psychiatry	204			< 0.001
1		20 (26%)	117 (92%)	
2		50 (65%)	9 (7.1%)	
3		7 (9.1%)	1 (0.8%)	

^*1*^ n (%); Median (Q1, Q3)

^*2*^ Pearson’s Chi-squared test; Wilcoxon rank sum test; Fisher’s exact test

By contrast, Cluster 2 is composed of 127 students, with a majority of students belonging to the online group (57%). The proportion of males is lower in this cluster (67%) and it presents a very high proportion of “agree” answers, indicating a strong satisfaction of the students in this group toward PBL.

The feature importance for cluster identification indicated that question 6 (PBL enhanced my understanding of child and adolescent psychiatry) and question 10 (I enjoyed learning about child and adolescent psychiatry via PBL) were the most important (S Fig. 9).

## Discussion

Our study aimed to compare the appreciation and outcomes of in-person and online PBL sessions in CAP. The findings indicate significant differences in the students’ perceptions and satisfaction levels between the two modalities, with implications for the future design and delivery of PBL.

### Principal findings

The primary observation was a statistically significant association between online delivery of PBL and higher overall student satisfaction, compared with the in-person cohort; given the historical-cohort design, this association is not interpreted as a causal effect of the modality. This was evidenced by the direct questionnaire responses (Table 2), the ordered logistic regression analysis, adjusted for age and sex (Table 3), and the results of the cluster analyses (Table 6). The composite scores derived from factor analysis further highlighted nuanced differences, with higher satisfaction levels in the online group for dimensions associated with questions 6 to 15, which focused on the perceived educational value and enjoyment of PBL (Fig. 2B).

The in-person group showed higher composite scores (*p* = 0.002; Table 4) which, given the reverse coding of the Likert scale (1 = Agree, 3 = Disagree), is consistent with lower satisfaction in that cohort and reconciles the item-level and composite analyses. In the adjusted multiple linear regression model, older age and male sex were also associated with higher composite scores. Cluster analysis reflected this trend, with in-person participants in the higher dissatisfaction cluster and online participants in the higher satisfaction cluster (Table 6).

### Interpretation of findings

The following interpretations are offered as hypotheses generated by, but not directly tested in, this observational comparison. They should be read as candidate explanatory mechanisms for the associations observed rather than as demonstrated causes. High satisfaction in the online PBL group aligns with literature on digital learning; co-incurrent effects like pandemic-related stressors may also have influenced both groups. Studies have noted that online platforms enable greater flexibility, accessibility, and personalized learning experiences [31, 32]. Previous qualitative studies have noted that online PBL can enhance flexibility, engagement, and access to resources, though initial adaptation may be challenging [33]. Our results further suggest that online PBL may enhance engagement and appreciation for topics like child and adolescent psychiatry, as reflected by the importance of questions 6 and 10 in cluster identification. In contrast, the lower satisfaction observed in the in-person cohort is consistent with reports of logistical constraints, rigid schedules, and limited opportunities for individual reflection in classroom-based PBL, although these mechanisms were not measured directly in the present study. Additionally, the fact that the PBL sessions were conducted in English might have contributed to the preference for online PBL, especially given the Japanese students’ potential need for additional time and resources to process and articulate their thoughts in a non-native language. This challenge is not unique to Japan and could similarly affect students from other non-English-speaking countries participating in English-medium PBL sessions. Online environments may have provided a more comfortable and less pressured setting, enabling students to review materials, formulate responses, and participate at their own pace. These findings are consistent with previous research highlighting the importance of learner autonomy and the role of language accessibility in PBL environments [34]. This is one of the reasons why authors of a systematic review about adoption of PBL in medical schools in non-western countries have recently recommended to proactively consider ways to adapt PBL education taking into account local contexts and culture [34]. Additionally, the observed pattern of correlated responses among certain questionnaire items (e.g., questions 1–3, 6–10, and 12–15) underscores the structured nature of PBL, where interconnected learning outcomes may influence students’ overall perceptions.

While our study was not designed to test theoretical frameworks and could not directly measure transactional distance, social presence, language anxiety or cognitive load, the observed differences between in-person and online PBL are compatible with the CoI [21] and TDT [20] frameworks summarised in the Background. In particular, the higher satisfaction observed in the online cohort would be consistent with reduced transactional distance (more time to process English-language content and to participate at one’s own pace) and with enhanced cognitive and teaching presence afforded by the flexibility of the online format, while any difficulties in spontaneous group interaction would be consistent with a relative limitation of social presence. When building the questionnaire, we divided it into 3 sections: the first section (questions 1 to 5) called “General impressions of PBL”, the second section (questions 6 to 10) called “Questions about PBL and child and adolescent psychiatry” and the third section (questions 11 to 16) called “Specific questions about the case”. The Factor analysis allowed us to understand that the second and third sections were included in a single dimension, which is consistent with their purposes. Four out of five questions included in the first section were included in the second dimension. The only question not linked to any dimension in this questionnaire was the fourth question: “PBL helps develop my communication skills”. Percentages of answers in each category of the Likert scale were extremely close among the two groups (*p* = 0.59 after adjustment) and were not statistically significantly impacted by neither age nor sex. A high proportion of students agreed with this question in both groups (69% in the in-person group and 67% in the online group). To enhance communication among healthcare professionals—a central objective of PBL [11]—it is essential to consider the challenges faced by students with high levels of communication apprehension. These students may experience significant difficulties participating in PBL sessions [35]. Therefore, when designing PBL scenarios and defining their implementation context, particular attention should be given to creating systems that facilitate communication opportunities for students with such apprehension.

Recent cross-cultural research using Hofstede’s cultural dimensions across medical students and trainees from sixteen different countries has shown that Japanese male medical students tend to score higher on power distance and long-term orientation, and lower on indulgence compared to their female counterparts [36]. Such profiles are compatible with a preference for structured, hierarchical environments among male students—more typical of traditional in-person formats—whereas the self-directed and egalitarian nature of online PBL may feel less aligned with their expectations; however, our design did not measure cultural dimensions directly and these mechanisms remain hypothetical. Additionally, lower indulgence scores suggest a more restrained approach to learning, which may reduce engagement with the flexible and autonomous aspects of online education. In contrast, female students—who scored lower on power distance and uncertainty avoidance, and higher on indulgence—may have found the online PBL format more conducive to open communication, autonomy, and enjoyment. These findings highlight the importance of considering gendered cultural profiles when designing and implementing PBL formats, especially in cross-cultural and digital learning environments.

### Implications for practice

These findings have several implications for teaching about CAP and medical education overall. First, in this cohort, online PBL was associated with higher student satisfaction, suggesting it is a viable modality, particularly for complex topics requiring active participation and reflection. Second, demographic factors such as age and gender should be considered in PBL design, with targeted interventions to address potential disparities. For example, younger students and females in our study showed relatively higher satisfaction. This observation aligns with broader cultural trends in Japan, where previous studies have reported that women often exhibit a heightened interest in foreign cultures and languages [37, 38]. Such interests might make them more receptive to English-language PBL, especially in an online format that allows greater exposure to and interaction with international educational methods. This trend underscores the need for tailored approaches to enhance engagement among older and male participants, who may not share the same level of intrinsic motivation for language and cultural immersion. One potential solution is to design scenarios in which the patient is a foreigner or the students are participating in internships in English-speaking countries, or Japanese tourists gets sick overseas. This approach enhances the sense of immersion and emphasizes the necessity of English [39]. Moreover, a study involving 48 Catalan/Spanish students who studied abroad demonstrated that they improved their English skills to a similar extent, regardless of whether they studied in an anglophone or non-anglophone country [40]. Thus, we propose that employing PBL in English for non-native English speakers could not only enhance medical education but also promote language acquisition. However, language barriers do not merely affect comprehension but also contribute to increased cognitive load, as suggested by Cognitive Load Theory (CLT) and the Cognitive Theory of Multimedia Learning (CTML). When learners struggle with the language of instruction, greater working memory resources are consumed in decoding linguistic content, leaving fewer resources available for processing and integrating new knowledge. This additional extraneous load can impair comprehension and limit meaningful participation in interactive activities such as PBL, where collaboration and communication are essential [41]. Collaboration between universities across different countries is likely to further increase student engagement and knowledge retention, as it exposes students to diverse perspectives, fosters cross-cultural communication, and creates opportunities for peer learning in a global context [42]. The new generation of students tends to be highly interested in cross-cultural communication and perceives digitalization as significantly simplifying these processes, particularly by helping to overcome language barriers [43]. These trends highlight the growing relevance of the intercultural competence framework, which emphasizes the development of attitudes, knowledge, and skills necessary for effective and respectful interaction across cultures—an essential component in preparing medical students for increasingly globalized and diverse learning environments [44]. At the same time, communication styles are strongly shaped by cultural contexts, which must be carefully considered when introducing collaboration between universities across different countries [45]. In parallel, insights from educational psychology highlight how digital technologies are reshaping learning processes, particularly by enabling training that adapts to individual learners’ sociocultural contexts, motivational states, and competencies. Beyond traditional knowledge acquisition, these technologies support the development of twenty-first-century skills such as self-regulated learning, collaborative problem-solving, and communication [46], which are increasingly essential in international and cross-cultural higher education collaborations. Ultimately, human-centered machine learning approaches have the potential to personalize learning content and adapt pacing to the needs of diverse learners, thereby enhancing engagement and reducing barriers to comprehension [47].

The slightly negative correlation (-0.09) observed between question 4 (“PBL helps to develop my communication skills”) and question 12 (“Mayumi’s case was well-written and understandable”) in the in-person group is compatible with greater challenges accessing translation tools in the classroom setting, although the present design cannot test this mechanism directly. However, this limitation does not appear to have diminished the effectiveness of PBL in enhancing their communication skills. A notable finding in our Factor Analysis was that Question 4 (‘PBL helps develop my communication skills’) did not load significantly onto either the ‘Perceived Learning Efficacy’ or ‘Engagement’ dimensions. While initially surprising, this isolation likely reflects the unique cognitive burden placed on Japanese medical students conducting PBL in English. For this demographic, communication is not merely a vehicle for learning but a distinct, high-stakes performance task fraught with ‘foreign language anxiety’. Consequently, students appear to compartmentalize ‘communication’ as a separate skill set, distinct from their appreciation of the medical content or the pedagogical method itself. This suggests that future evaluations of PBL in cross-cultural settings should treat linguistic communication as an independent domain of assessment, rather than subsuming it under general engagement. The most important questions in order to be classed in the cluster analysis were questions 6 (PBL enhanced my understanding about child and adolescent psychiatry) and question 10 (I enjoyed learning about child and adolescent psychiatry via PBL). This suggests a strong preference among participants for engaging, enjoyable learning experiences, highlighting the importance of ludic elements (i.e., game-like or playful components that enhance motivation and engagement) in education [48]. This is consistent with the results of a Brazilian study on 106 veterinary medicine students, which showed that gamed-based learning allows knowledge acquirement in the same scale as case-based learning (CBL) and peer tutoring, but also promotes a greater perception of the stimulus for self-study and problem-solving ability and contributes to the development of group dynamics compared with the group who received CBL (*P* < 0.05) [49].

### Strengths and limitations

Our study is among the first to employ both factor analysis and clustering to evaluate PBL satisfaction comprehensively. The inclusion of multiple statistical approaches, including logistic regression, linear regression, factor analyses and cluster analyses, adds robustness to our findings. Additionally, the use of a standardized questionnaire allowed for detailed exploration of student perceptions across different dimensions.

However, several limitations should be acknowledged. First, the study was conducted at a single institution with a culturally homogeneous sample, so caution should be exercised when generalizing these findings beyond Nagoya or the Japanese context. Additionally, the study did not assess the English proficiency of participants prior to the PBL sessions, which limits our ability to directly quantify how language skills influenced comprehension, interaction, and satisfaction. Research indicates that Japanese students often face challenges in expressing themselves and comprehending content in English due to differences in linguistic structures and limited opportunities for conversational practice [17]. These factors may have contributed to the findings. To address these issues, future implementations should consider providing tailored vocabulary lists, glossaries, and other language aids designed to align with the specific PBL topics and collaborate with international universities. Encouraging participation through structured prompts and fostering a supportive, low-pressure environment may also help mitigate language barriers and enhance the inclusivity of PBL sessions. Third, the cross-sectional design precludes causal inference, and longitudinal studies are needed to assess the long-term impact of PBL modalities on educational outcomes. Fourth, this study has limitations inherent to its naturalistic design, including the comparison of pre-pandemic (2019) and pandemic-era (2021) cohorts. Because of the design of the study, no randomization was done and consequently observed differences should be interpreted as associative rather than causal. Fifth, the questionnaire used in this study was adapted from a previously reported instrument and reviewed by faculty experts, but it did not undergo a separate pilot reliability study before the 2019 administration. Although the KMO statistic, Bartlett’s test, parallel analysis, Cronbach’s alpha values and the WLSMV-based ordinal CFA reported above support the two-factor structure and the internal consistency of the scale, in the absence of an a-priori pilot study these psychometric results should be regarded as part of the on-going validation of the instrument rather than as a confirmation of pre-established validity. Sixth, although the item-level ordered-logistic comparisons reported in Table 3 carry small unadjusted *p*-values, they were not formally corrected for multiplicity and should be interpreted as exploratory descriptors of which questionnaire items contributed to the global modality effect; replication in larger, prospective samples is needed before they are taken to support item-specific inference.

The psychological, social, and academic disruptions caused by the pandemic may have influenced the 2021 cohort’s appreciation for online PBL, potentially inflating satisfaction due to the availability of continued education during a crisis rather than reflecting a clear preference for the online format itself.

Additionally, variables such as increased exposure to digital tools, variable home learning conditions, and changes in institutional teaching practices during the pandemic complicate the interpretation of results. Moreover, the use of a three-point Likert scale in the 15-item survey may have limited the granularity of responses and introduced potential ceiling or floor effects, reducing the sensitivity to detect subtle differences in student perceptions. We can also note that question 4, intended to assess the development of communication skills, did not load significantly on any factor in the exploratory factor analysis. This indicates that our questionnaire may not fully capture this critical learning outcome, and future studies should refine or expand items to better assess communication skills development in PBL. Nevertheless, this study’s findings remain relevant and supported by comparable work conducted both during and outside the pandemic. For instance, another study conducted during the pandemic demonstrated enhanced student satisfaction and perceived effectiveness of virtual PBL despite challenges in transitioning [50]. Similarly, a Brazilian study, who examined during the pandemic hybrid formats, reported that well-structured online PBL—with appropriate facilitation—continued to foster student engagement and learning outcomes [51]. Donkin et al. conducted a scoping review of 13 studies on online Case-Based Learning (CBL) in medical education published between 2010 and 2022, including 9 studies during the COVID-19 pandemic and 4 from before [52]. Given the pedagogical similarities between CBL and Problem-Based Learning (PBL)—both relying on collaborative, student-centered case analysis—the findings are highly relevant. The review showed that online formats maintained or improved learning outcomes, despite challenges like communication barriers and internet access. While replication in non-pandemic contexts is needed, our findings contribute to a growing consensus that hybrid or online PBL can address demographic and linguistic barriers in global medical education.

### Future directions

Future research should focus on fostering collaborations between universities from different countries to implement and study PBL among non-native English-speaking populations. Such initiatives could significantly boost students’ interest and participation by exposing them to diverse educational practices and cultural perspectives. Cross-cultural studies involving diverse student populations are essential to validate our findings and refine PBL strategies to accommodate different linguistic and cultural contexts. Educators transitioning from face-to-face to online PBL in multicultural settings should consider implementing step-by-step strategies such as: providing bilingual materials and glossaries, incorporating culturally relevant case scenarios, and offering structured prompts to support participation. These approaches can help mitigate language barriers and foster inclusive engagement. Finally, additional efforts should aim at enhancing critical thinking and addressing demographic disparities in PBL satisfaction. As the majority of medical literature is published in English [53], proficiency in this language is essential for future medical doctors. It enables them to critically assess the quality and reliability of their sources, ensuring that their clinical decisions are based on the best available evidence. Moreover, English proficiency allows medical professionals to engage with the latest advancements in the field, participate in international collaborations, and continuously update their knowledge throughout their careers, fostering lifelong learning and evidence-based practice.

## Conclusion

Our findings are consistent with an association between online delivery of PBL and higher student satisfaction in this medical education setting, particularly for topics requiring high engagement and reflection; given the observational, two-cohort design, this association should not be interpreted as a causal effect. By leveraging digital tools and addressing demographic factors, educators can optimize PBL experiences to meet the evolving needs of students. These insights contribute to the growing body of evidence supporting innovative approaches to medical education and highlight the importance of tailoring PBL to diverse learning environments.

## Supplementary Information

Supplementary Material 1.

Supplementary Material 2.

Supplementary Material 3.

Supplementary Material 4.

Supplementary Material 5.

Supplementary Material 6.

Supplementary Material 7.

Supplementary Material 8.

Supplementary Material 9.

Supplementary Material 10.

Supplementary Material 11.

## Data Availability

The datasets used and/or analyzed during the current study are available from the Office of International Affairs, Nagoya University Graduate School of Medicine, upon reasonable request. Requests can be directed to: med-intl(at)t.mail.nagoya-u.ac.jp.
