# Supplementary material for: Comparing traditional and online problem-based learning in child and adolescent psychiatry in Nagoya: a novel statistical approach in Japanese educational settings
Source: BMC Med Educ. 2026 Jun 10;26:1328. doi: 10.1186/s12909-026-09517-9 (PMC13483616; doi:10.1186/s12909-026-09517-9)
Supplement: Supplementary file 1 [file 12909_2026_9517_MOESM1_ESM.docx]

**Questionnaire**

**Students' perceptions about Problem Based Learning in child and adolescent psychiatry**

Please do not write your name or surname.

Age…….

Gender……

Nationality …..

*Please provide your opinion about PBL in child and adolescent psychiatry at Nagoya University (NU)*

|  |  | **Agree** | **Neutral** | **Disagree** |
| --- | --- | --- | --- | --- |
| **A** | **General Questions about PBL at the NU** |  |  |  |
| 1 | PBL helps me to develop skills to solve clinical problems |  |  |  |
| 2 | PBL is a good way of learning the content of the course |  |  |  |
| 3 | PBL encourages my self-directed learning |  |  |  |
| 4 | PBL helps to develop my communication skills |  |  |  |
| 5 | I think, PBL is more effective way of learning compared to traditional lecture |  |  |  |
|  | | | | |
| B | **Questions about PBL in child & adolescent psychiatry** |  | | |
| 6 | PBL enhanced my understanding about child & adolescent psychiatry |  |  |  |
| 7 | PBL increased my interest in child & adolescent psychiatry |  |  |  |
| 8 | Child and adolescent psychiatry because of it’s emphasis on teamwork and collaboration, would be a specialty learned optimally through PBL |  |  |  |
| 9 | Duration of PBL session was enough to learn about child & adolescent psychiatry |  |  |  |
| 10 | I enjoyed learning about child and adolescent psychiatry via PBL |  |  |  |
|  | | | | |
| **C.** | **Specific questions about case “Mayumi” (Adolescent depression).** | | | |
| 11 | I think Mayumi’s case is common for a clinical-settings in Japan. |  |  |  |
| 12 | Mayumi’s case was well written and understandable |  |  |  |
| 13 | Mayumi’s case has an interesting clinical trigger |  |  |  |
| 14 | Mayumi’s case has an appropriate level of difficulty/challenge |  |  |  |
| 15 | Mayumi’s case helped me to understand the importance of multidisciplinary approach in child and adolescent psychiatry |  |  |  |

Do you have other thoughts or recommendations how to improve PBL in child and adolescent psychiatry? (Use other side if needed to provide your answer)
