# Supplementary material for: Comparing traditional and online problem-based learning in child and adolescent psychiatry in Nagoya: a novel statistical approach in Japanese educational settings
Source: BMC Med Educ. 2026 Jun 10;26:1328. doi: 10.1186/s12909-026-09517-9 (PMC13483616; doi:10.1186/s12909-026-09517-9)
Supplement: Supplementary file 2 [file 12909_2026_9517_MOESM2_ESM.docx]

| **S. Table 2. Statistical methods used and their purposes** | |
| --- | --- |
| **Statistical Method** | **Purpose** |
| Composite Score Development | To quantify multidimensional aspects of student outcomes using all questionnaire items |
| Linear Regression | To assess whether composite scores differed by PBL group, adjusting for age and sex |
| Ordered Logistic Regression | To evaluate individual Likert responses while adjusting for demographic variables |
| Visualization of Relationships | To explore correlations and patterns among questionnaire items |
| Factor Analysis | To identify latent dimensions of student engagement and validate item groupings |
| Cluster Analysis | To classify students into subgroups based on response patterns and satisfaction levels |
